# Surface crystal feature-dependent photoactivity of ZnO–ZnS composite rods *via* hydrothermal sulfidation†

**DOI:** 10.1039/c7ra13061a

**Published:** 2018-01-31

**Authors:** Yuan-Chang Liang, Chein-Chung Wang

**Affiliations:** Institute of Materials Engineering, National Taiwan Ocean University Keelung 20224 Taiwan yuanvictory@gmail.com

## Abstract

ZnO–ZnS core–shell composite rods were synthesized using a two-step facile hydrothermal methodology wherein different sulfidation durations were employed. The effects of sulfidation duration on the morphology and crystalline quality of ZnS shell layers on the surfaces of ZnO rods were investigated. A ZnS shell layer with visible granular features was obtained in the adequately controlled 3 h sulfidation process. A structural analysis demonstrated that the ZnS shell layers of ZnO–ZnS composite rods synthesized after 3 h sulfidation were in a well-defined crystalline cubic zinc blend phase. Moreover, optical properties revealed that these composite rods had a higher light-harvesting ability than those obtained after 1 and 2 h sulfidation. The density of surface crystal defects and the photoexcited charge separation efficiency of the composite rods were associated with changes in the microstructure of the synthesized ZnS shell layers. The optimal sulfidation duration of 3 h for the ZnO–ZnS composite rods resulted in the highest photocatalytic activity for the given photodegradation test conditions. The improved light harvesting and charge transport at the ZnO–ZnS heterointerface accounted for the enhanced photocatalytic activity of the ZnO–ZnS composite rods synthesized after 3 h sulfidation.

## Abstract 2

ZnO–ZnS core–shell composite rods were synthesized using a two-step facile hydrothermal methodology wherein different sulfidation durations were employed.

## Graphical abstract

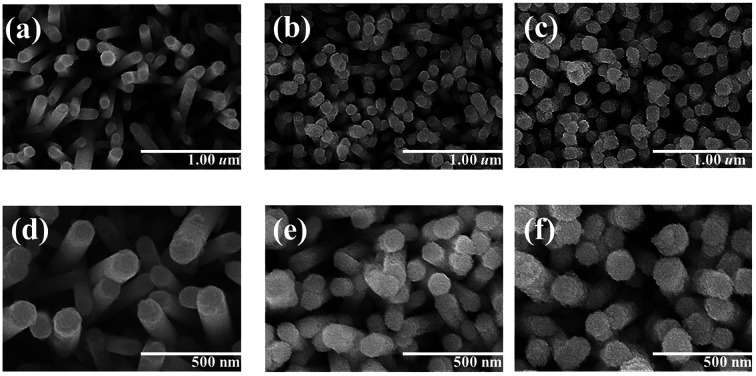

## Introduction

The development of oxide semiconductor photocatalysts for environmental remediation has been widely studied.^1–3^ Zinc oxide (ZnO) has received global research interest for application as a highly efficient photocatalyst. However, low quantum efficiency, short-range disorder, and photocorrosion continue to affect the performance of ZnO in practical applications.^4,5^ Several studies have been proposed to enhance the properties of ZnO by covering the surface with another semiconductor.^6–10^ Moreover, formation of a heterostructure is beneficial to improve the photocorrosion effect of ZnO.^11,12^ Among various heterostructured systems, the core–shell system, where the shell plays a crucial role as a physical barrier between the optically active core and the surrounding medium, was proposed for ZnO-based composites.^6,13^ In most of the studies, heterogeneous core–shell structures were formed by oxide–oxide and oxide-sulfide composites. Ruosong *et al.*, reported the hydrothermal synthesis of ZnO–TiO_2_ core–shell nanorods for photocatalysts and investigated the photocatalytic decomposition of rhodamine B. An enhanced photocatalytic activity was observed for ZnO–TiO_2_ compared with pure ZnO.^14^ Moreover, Guifeng *et al.*, showed that ZnO–CdS core–shell nanowires exhibit an enhanced light-harvesting ability in both UV and visible light ranges. The increased shell surface roughness led to the improved photocatalytic decomposition of methylene blue (MB) compared with bare ZnO.^15^ ZnO–Ag_2_S core–shell nanoparticles synthesized using a low-temperature chemical method demonstrated a superior photocatalytic activity than ZnO nanoparticles at both acidic and basic pH values.^16^ These results demonstrate that integrating oxide-sulfide to form a ZnO-based core–shell composite is promising to improve the light-harvesting properties of ZnO and enhance the photocatalytic reaction efficiency.

Zinc sulfide (ZnS) is an important semiconductor, and nanostructured ZnS offers unique optical and catalytic properties. It is nontoxic and water insoluble.^17^ In addition, ZnS is an effective photocatalyst because of the rapid generation of electron–hole pairs by photoexcitation and the highly negative reduction potentials of the excited electrons. However, the band gap of ZnS is large and some studies have reported that its band gap can reach up to 3.66 eV,^18^ which is extremely large and thus decreases the light-harvesting ability of ZnS. In this regard, a core–shell heterogeneous structure was an innovative strategy for designing highly efficient photocatalysts incorporated with ZnS. However, another important factor that affects the photocatalytic performance is the band alignment between core and shell materials. A type-II band alignment is suitable for an efficient charge separation between core and shell semiconductors. Based on the aforementioned discussions, the integration of ZnS into ZnO to form a ZnO-based core–shell heterostructure is a promising approach for improving the photocatalytic performance of both ZnO and ZnS. However, only a few studies have reported the synthesis and photocatalytic applications of such ZnS–ZnO core–shell composites. In the present study, a ZnO–ZnS core–shell structure was synthesized using a facile two-step hydrothermal methodology. The crystalline feature of the constructed shell layer generally has a profound effect on the photocatalytic performance of core–shell composites. However, this topic has rarely been investigated for ZnO–ZnS core–shell composites synthesized in a hydrothermal sulfidation reaction. The photocatalytic decomposition of MB dyes by using ZnO–ZnS core–shell composites hydrothermally derived at different sulfidation durations was studied. The correlation between the structure and the optical and photocatalytic performance of these heterogeneous ZnO–ZnS core–shell composites was discussed.

## Experiments

In this study, ZnO–ZnS core–shell composite nanorods with different ZnS shell layer crystal qualities were fabricated. Hydrothermally synthesized high density ZnO nanorods on the 200 nm-thick SiO_2_/Si (100) substrates were used as templates for further sulfidation treatment to grow ZnO–ZnS core–shell nanorods. The synthesis of vertically aligned ZnO rods consisted of two steps corresponding to the formation of ZnO seed layer and the growth of rods. The detailed experiment on the synthesis of hydrothermally synthesized ZnO rods has been described elsewhere.^19^ Theas-synthesized ZnO rods were immersed in a Teflon autoclave containing 0.05 M thioacetamide (TAA) aqueous solution. The reaction system was heated to 130 °C and kept for 1, 2, and 3 h to grow ZnO–ZnS core–shell rods. The ZnO–ZnS-1, ZnO–ZnS-2, and ZnO–ZnS-3 are used to present the as-synthesized ZnO–ZnS core–shell rods after 1, 2, and 3 h sulfidation, respectively in this study. Finally, the reaction system was cooled to room temperature naturally, and then the final material was washed in deionized water and dried in an oven.

Crystal structures of the as-synthesized ZnO–ZnS core–shell rods were investigated by X-ray diffraction (XRD) using Cu Kα radiation. The surface morphologies of the various samples were characterized by scanning electron microscopy (SEM), and high-resolution transmission electron microscopy (HRTEM) was used to investigate the detailed microstructures of the core–shell rods. Room-temperature-dependent photoluminescence (PL) spectra were obtained using the 325 nm line of a He–Cd laser. The analysis of absorbance spectra of the core–shell rods were conducted by using UV-Vis spectrophotometer. To measure photocurrent properties of the samples, silver glues were laid on the surfaces of the rods to form two contact electrodes and the applied voltage was fixed at 5 V during electric measurements under irradiation. Photocatalytic activity of various ZnO–ZnS rod samples were performed by comparing the degradation of aqueous solution of methylene blue (MB; 5 × 10^−6^ M) containing various ZnO–ZnS rods as catalysts under solar light irradiation excited from the 100 W Xe arc lamp. The solution volume of MB is 20 ml and the sample size for tests has a fixed coverage area of 1.0 cm × 1.0 cm for photocatalytic test use.

## Results and discussion

**Fig. 1:**
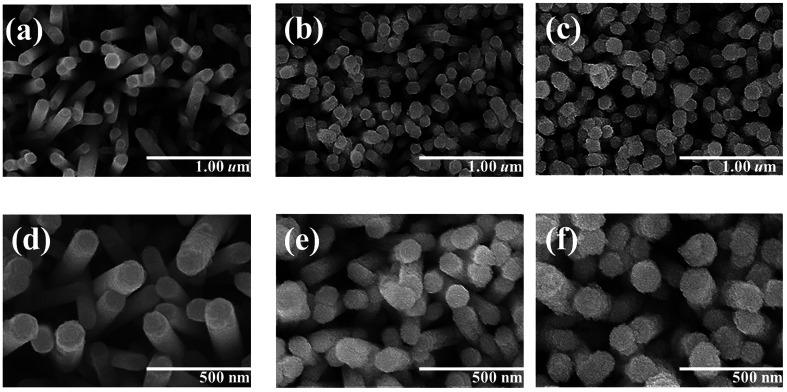
Low-magnification SEM images of ZnO–ZnS composite rods with various sulfidation durations: (a) ZnO–ZnS-1, (b) ZnO–ZnS-2, and (c) ZnO–ZnS-3. The corresponding high-magnification SEM images are shown in (d), (e), and (f), respectively.

Fig. 1(a)–(c) show low-magnification SEM images of the ZnO rods sheathed by the ZnS layer; the composite rods were obtained at three hydrothermal sulfidation durations (1, 2, and 3 h). Fig. 1(d)–(f) display the corresponding high-magnification SEM images. The SEM images indicate that the surface of the composites changed with the sulfidation duration. As shown in Fig. 1(a) and (d), the smooth surface of the hexagonal ZnO rods turned into a rough surface after 1 h sulfidation. Layered aggregates homogeneously covered the ZnO rods and the hexagonal faces of the ZnO rods became rounder after sulfidation. These observations suggested the partial conversion of ZnO into ZnS, thus leading to a composite structure. When the sulfidation duration was further increased to 2 h, the surface of the ZnO–ZnS rods became rougher than that of the rods subjected to only 1 h sulfidation (Fig. 1(b) and (e)). The surface of the layered aggregates of the composite rods became rougher after 2 h sulfidation. Fig. 1(c) and (f) show that the surface of the composite rods obtained after 3 h sulfidation was granular. This might be associated with an increased crystallization of the surface ZnS crystallites with the increasing sulfidation duration in the hydrothermal synthesis process. Notably, increasing the sulfidation duration to 4 h caused consumption of the ZnO rods and the formation of ZnS tube-like features (Fig. S1†).

**Fig. 2:**
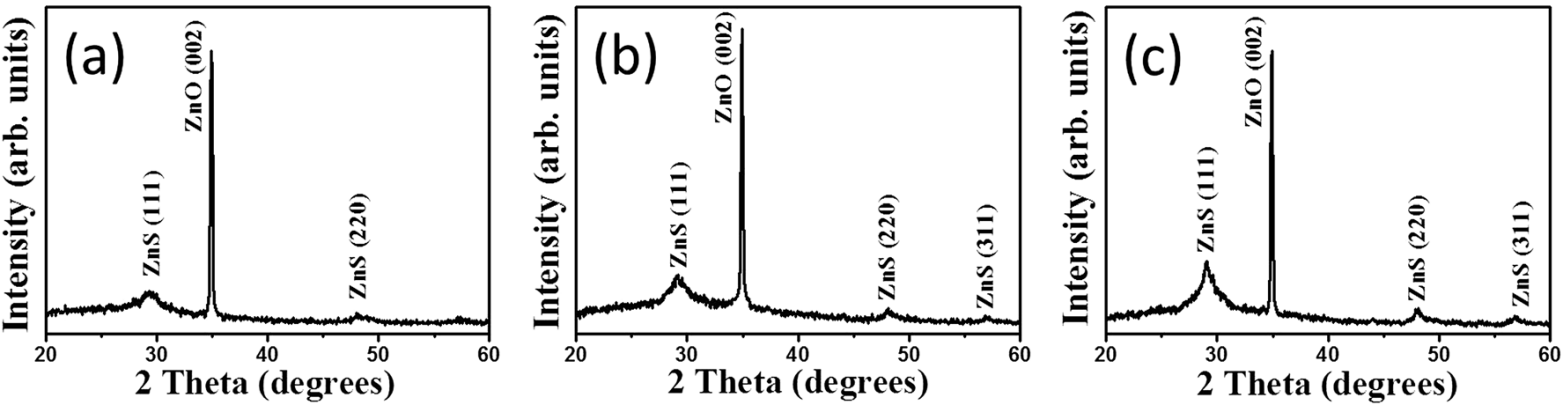
XRD patterns of ZnO–ZnS composite rods with various sulfidation durations: (a) ZnO–ZnS-1, (b) ZnO–ZnS-2, and (c) ZnO–ZnS-3.

Fig. 2(a) shows XRD pattern of ZnO–ZnS-1 rods. An intense peak located at approximately 34°, which is corresponded to the ZnO (002) plane (JCPDS no. 36-1451). In addition, no peaks except the (00*l*) Bragg reflections are observed in the whole measurement range, indicating the highly *c*-axis oriented hexagonal ZnO rods were used for sulfidation processes. Moreover, several tiny and broaden Bragg reflections centered at approximately 29.4° and 48.1° corresponding to (111) and (220) Bragg reflections of cubic zinc blend ZnS, respectively (JCPDS no. 05-0566) were visible in the XRD pattern. This reveals that ZnS crystallites were formed on the surfaces of ZnO rods after 1 h sulfidation. Fig. 2(b) shows the XRD pattern of ZnO–ZnS-2 rods. The intensity of ZnS (111) and (220) Bragg reflections increased and ZnS (311) Bragg reflection became visible. Further increased sulfidation duration to 3 h (Fig. 2(c)), all the Bragg reflections from ZnS became more intense and the full-width at half maximum of the ZnS Bragg reflections became narrower compared with those ZnO rods treated with shorter sulfidation duration. Moreover, the Bragg reflection originated from ZnO rods is still intense after 3 h sulfidation. Based on the XRD observations, the ZnO–ZnS composites were formed when the ZnO rods were subjected to sulfidation processes with various durations (1 to 3 h) and the crystallinity of the as-formed ZnS layer was improved with increased sulfidation duration.

**Fig. 3:**
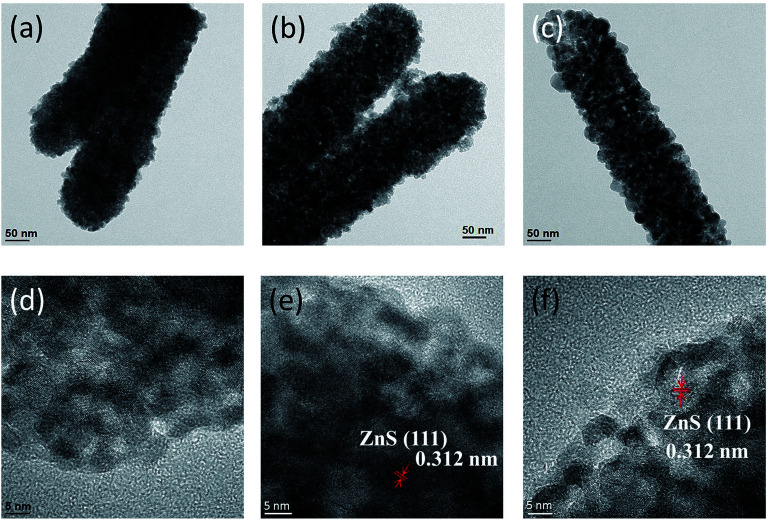
Low-magnification TEM images of ZnO–ZnS composite rods with various sulfidation durations: (a) ZnO–ZnS-1, (b) ZnO–ZnS-2, and (c) ZnO–ZnS-3. The corresponding high-resolution images obtained from the edge of the ZnS shell layer are shown in (d), (e), and (f), respectively.

Fig. 3(a)–(c) present low-magnification TEM images of the ZnO–ZnS composite rods synthesized with different sulfidation durations. With 1 h sulfidation, the ZnS shell layer appeared to be composed of loose sponge-like aggregates, as shown in Fig. 3(a). With an increase in the sulfidation duration, particle-like aggregates of the ZnS shell layer became more predominant (Fig. 3(b)). When the sulfidation duration reached 3 h (Fig. 3(c)), the ZnS shell layer structure exhibited a highly granular surface with a distinguishable particle size and boundaries with adjacent grains. The ZnO–ZnS composite rods synthesized with 1 h sulfidation exhibited a smoother surface than the composite rods synthesized with higher sulfidation durations. Fig. 3(d) presents a high-resolution TEM image of the outer region of the ZnS shell for the ZnO–ZnS-1 composite rod. Ordered lattice fringes were distinguishable and distributed within a short range of the selected region in the ZnS shell layer synthesized after 1 h sulfidation. Furthermore, grain boundaries and ordered lattice fringes with an interval of approximately 0.312 nm were visible and assigned to the (111) plane of cubic ZnS, as shown in Fig. 3(e)–(f). This observation verifies the previously reported structure of hydrothermally derived cubic ZnS crystallites.^20^ The TEM analyses revealed that the ZnS shell layer synthesized after extending the sulfidation duration from 1 h to 3 h exhibited an improved crystallization and an increased particle size. These observations are consistent with the XRD results.

**Fig. 4:**
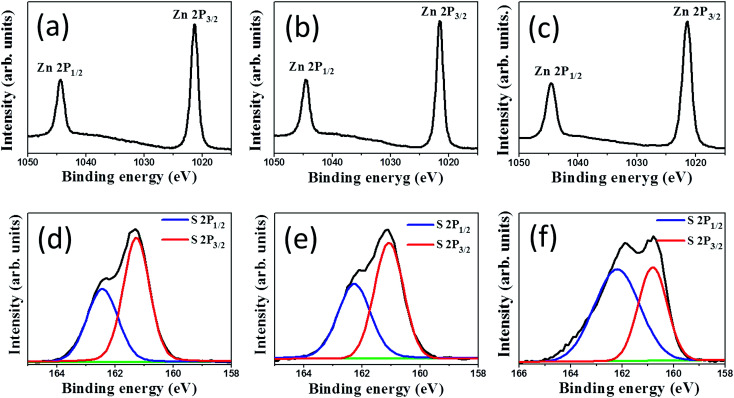
High-resolution XPS spectra in Zn 2p region of ZnO–ZnS composite rods with various sulfidation durations: (a) ZnO–ZnS-1, (b) ZnO–ZnS-2, and (c) ZnO–ZnS-3. High-resolution XPS spectra in S 2p region of ZnO–ZnS composite rods with various sulfidation durations: (d) ZnO–ZnS-1, (e) ZnO–ZnS-2, and (f) ZnO–ZnS-3.

The difference in the chemical binding status of the ZnS shell layers synthesized with different durations of sulfidation of ZnO rods was examined through XPS measurements. Fig. 4(a)–(c) show the XPS surface narrow scan Zn 2p spectra of the ZnO–ZnS composite rods synthesized with 1, 2, and 3 h sulfidation, respectively. In the Zn spectrum, the peaks at binding energies of 1044.4 and 1021.3 eV are assigned to the Zn 2p_1/2_ and Zn 2p_3/2_ peaks of Zn^2+^, respectively.^21^ Moreover, the Zn 2p doublet separations in Fig. 4(a)–(c) appeared at 23.0–23.1 eV, which is in good agreement with previously reported values for zinc ions binding to sulfur ions in ZnS lattices.^22^ The asymmetric S 2p peak in Fig. 4(d)–(f) was deconvoluted into two subpeaks corresponding to S 2p_3/2_ and S 2p_1/2_ and were located at 161.1 and 162.2 eV, respectively. The binding energy of 161.1 eV originated from S^2−^ in the ZnS structure. The subpeak at approximately 162.2 eV might be due to surface defects of the S–S species in the ZnS shell layer and was previously reported in chemically derived ZnS nanorods.^23^ Notably, the intensity ratio of the spin–orbit splitting peaks for S 2p_3/2_ and S 2p_1/2_ was approximately 2 : 1 for the ZnS shell layers after 1 and 2 h sulfidations. This is in agreement with previous reports.^24^ However, the intensity ratio of the spin–orbit splitting peaks for S 2p_3/2_ and S 2p_1/2_ was determined to be approximately 1 : 1 for the ZnS shell layer after 3 h sulfidation. This can be attributed to the increased bonding states of sulfur-related surface defects in ZnS.^25^

**Fig. 5:**
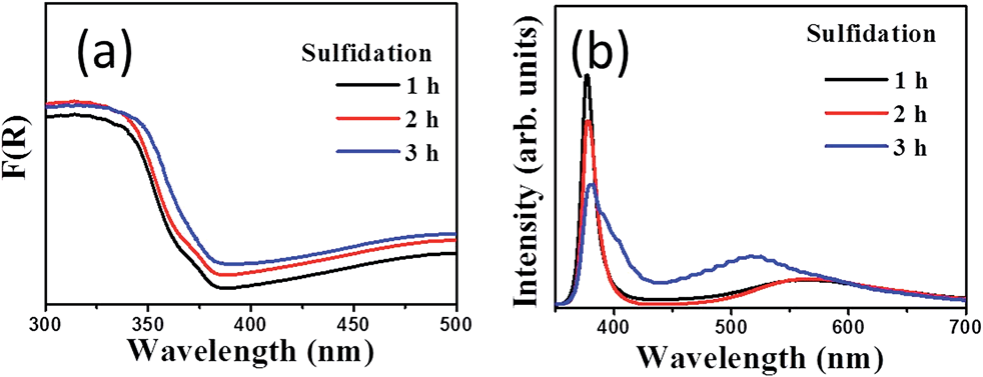
(a) Optical absorbance spectra of various ZnO–ZnS composite rods. (b) PL spectra of ZnO–ZnS composite rods with various sulfidation durations.

Fig. 5(a) compares the optical absorption curves of ZnO–ZnS core–shell rods obtained with different sulfidation durations. The absorbance band edges of the ZnO–ZnS-1 and ZnO–ZnS-2 composites rods were located in the UV region because both ZnO and ZnS compounds had band gap energies in the UV region. However, the absorbance band edge of the ZnO–ZnS-3 rods was slightly red-shifted. The slightly improved light-harvesting ability of the ZnO–ZnS composites formed after 3 h sulfidation may be a consequence of the increase in the formation of surface defect centers in the ZnS shell layer and the formation of surface granule-like layer features, which increased light scattering.^6^ In microwave-assisted surface sulfidation synthesis of ZnO–ZnS heterostructured microflowers, the introduction of more S^2−^ ions during synthesis creates a higher number of surface defect centers and, consequently, improved light harvesting and photocatalytic activity.^26^ Furthermore, an increase in the ZnS particle load on ZnO in the chemical bath synthesis of ZnO–ZnS nanowires showed an increased electronic interaction between ZnO and ZnS, and therefore a pronounced red shift of the edge of the absorbance band.^27^Fig. 5(b) shows the PL spectra of the ZnO–ZnS composite rods obtained with different sulfidation durations. In Fig. 5(b), the ZnO–ZnS-1 and ZnO–ZnS-2 composite rods have similar PL spectra. A sharp UV emission band centered at 378 nm and a distinct broad green emission band centered at 567 nm were clearly visible. Furthermore, the PL peak in the UV region was still present for the ZnO–ZnS-3 rods, but an additional peak in the blue region and a visible light emission band centered at 520 nm appeared. The peak in the PL spectrum of the ZnO–ZnS-3 composite rods differed from that of the composite rods obtained after 1 and 2 h sulfidation. The UV emission band was attributed to the nearest band edge emission originating from the recombination of free excitons of ZnO through an exciton–exciton collision process.^2,6^ The green emission band centered at 567 nm was previously attributed to the contribution of the surface defects in ZnO.^2^ Notably, the origin of the green emission centered at 520 nm for the ZnO–ZnS-3 composite rods be assigned to the deep states from Zn vacancy in the ZnS crystallites. By contrast, the emission band in the blue region was due to the sulfur vacancy and interstitial lattice defects in the ZnS nanostructures.^28^ The difference in the PL spectrum for the ZnO–ZnS rods synthesized with 3 h sulfidation might be associated to the sulfidation duration-dependent crystal quality differences in the ZnS shell layers. The ZnS shell layer formed after 3 h sulfidation showed clear granule-like features and the crystalline quality increased with the sulfidation duration. The prolonged crystal growth due to an increased duration of the hydrothermal sulfidation reaction might create more point defects in the solid-state ZnS crystallites. The aforementioned reasons might account for the PL spectrum of the ZnO–ZnS composite rods obtained after 3 h sulfidation being different from those of the composite rods obtained with a shorter sulfidation time. According to the PL spectra, the intensity of the PL peak in the UV region clearly decreases when the sulfidation duration increases. The decrease in the intensity of the UV emission band could be attributed to the different heterointerface states of band alignment between ZnO and ZnS.^29^ Moreover, our results show that the ZnO–ZnS composite rods had a higher charge separation efficiency for a prolonged sulfidation duration.

**Fig. 6:**
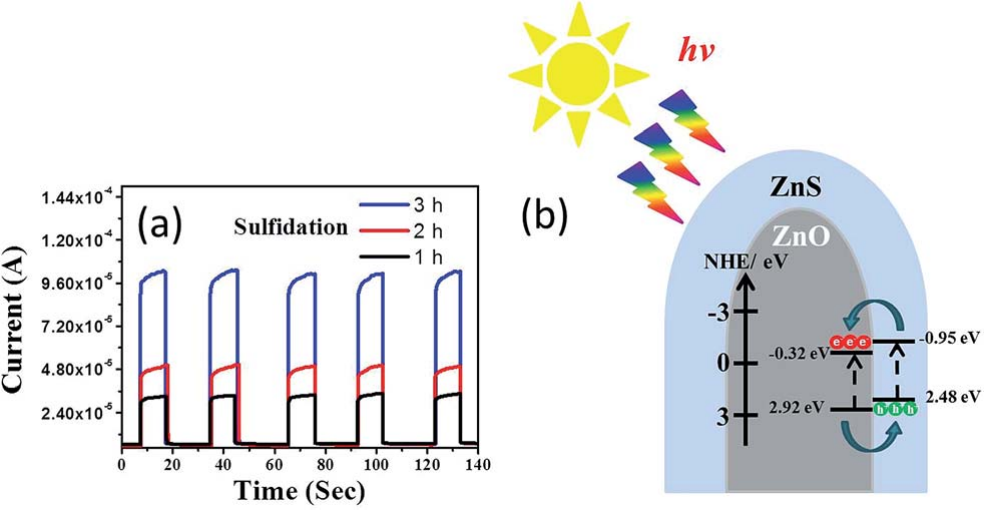
(a) Photocurrent *vs.* time plot for ZnO–ZnS composite rods with various sulfidation durations under chopped irradiation. (b) The schematics of band alignment and charges transfer at the ZnO–ZnS interface.

The photoresponses of the ZnO–ZnS composite rods obtained with various sulfidation durations were also investigated to determine the charge separation efficiency of photoexcited carriers at the ZnO–ZnS heterointerface. The corresponding photocurrent responses to several light cycles for the ZnO–ZnS composite rods obtained with different sulfidation durations are shown in Fig. 6(a). The fast and uniform photocurrent responses suggested a quick and stable charge transport in the samples. In the dark, the measured current for the ZnO–ZnS composite rods obtained with various sulfidation durations were low and were approximately 5.7 × 10^−6^, 6.1 × 10^−6^, and 6.4 × 10^−6^ A, respectively. Under light irradiation, the photocurrent increased sharply to reach a value as high as 3.2 × 10^−5^, 5.2 × 10^−5^, and 1.0 × 10^−4^ A for each prepared sample. Moreover, it decreased quickly as soon as the light was turned off. The photocurrent responses showed that the ZnS–ZnO composite rods obtained after 3 h sulfidation achieved a 15-fold increase in current when exposed to light irradiation. This increase was higher than that for the ZnO–ZnS composite rods synthesized with 1 h (5.6-fold increase) and 2 h (8.5-fold increase) sulfidation. Fig. 6(b) presents the band alignment of the ZnO–ZnS heterostructure.^30^ The mechanism underlying the photoresponse of the ZnO–ZnS composite rods could be explained through the photogenerated electron–hole pairs in the ZnS shell layer. The pairs separate and electrons are injected into the conduction band of the ZnO core. Moreover, the photogenerated holes in the ZnO core are transferred to the valence band of the ZnS shell because of the type-II band alignment in the ZnO–ZnS system.^30^ A similar band alignment of the oxide-sulfide heterostructures for improved photoactivity was observed in WO_3_–CdS.^31^ The relatively high photoresponse level of the ZnO–ZnS composite rods obtained with 3 h sulfidation in this study implies a fast separation and transport of the photogenerated holes and electrons at the ZnO–ZnS heterointerface.

**Fig. 7:**
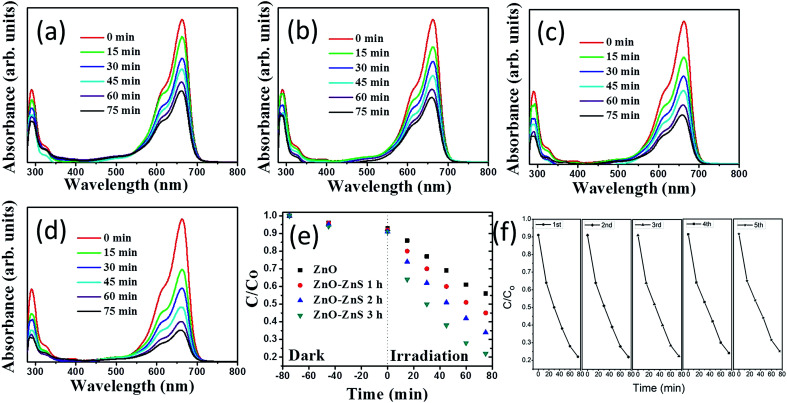
Intensity variation of absorbance spectra of the MB solution *vs.* irradiation duration containing various ZnO–ZnS rod samples under solar light illumination: (a) pristine ZnO rods. (b) ZnO–ZnS-1. (c) ZnO–ZnS-2. (d) ZnO–ZnS-3. (e) *C*/*C*_o_*vs.* irradiation time curves for the MB solution containing various ZnO–ZnS rod samples in dark conditions and under solar light illumination. (f) Recycled performances in the presence of the ZnO–ZnS-3 for photodegradation of MB dyes.

The photoactivity of the ZnO–ZnS composite rods obtained with different sulfidation durations was evaluated through the catalytic degradation process of an aqueous MB solution under solar irradiation. Fig. 7(a)–(d) show the absorbance spectra of the MB solution containing ZnO rods and various ZnO–ZnS composite rods under solar irradiation for various durations. The intensity of the absorbance peaks gradually decreased with increasing exposure time. This result indicates that MB is gradually degraded under the light irradiation in the presence of ZnO rods and ZnO–ZnS composite rods. The decrease in the absorbance peaks in both the UV and visible regions was more intense for the MB solution containing the ZnO–ZnS composite rods than for the MB solution containing the ZnO rods. Moreover, a relatively higher decrease in the intensity of the absorbance peak of the MB solution containing various ZnO–ZnS composite rods was observed in both the UV and visible regions for the composite rods obtained with 3 h sulfidation for a given irradiation time. The variation in the intensity of the absorbance peaks centered at 663 nm was further used to evaluate the photoactivity of various ZnO–ZnS composite rods. The photodegradation level of the MB solution containing various ZnO–ZnS composite rods is defined as (*C*/*C*_o_), where *C*_o_ is the concentration of aqueous MB without irradiation after the dark adsorption equilibrium and *C* is the concentration of aqueous MB corresponding to a given solar light irradiation duration.^32^Fig. 7(e) displays the value of *C*/*C*_o_ as a function of the irradiation duration for MB solutions containing ZnO rods and ZnO–ZnS composite rods obtained with different sulfidation durations. From Fig. 7(e), all of the ZnO–ZnS composite rod photocatalysts achieved the discoloration of the MB solution and showed relatively higher photocatalytic activities than the ZnO rods. The photodegradation level after a 75 min irradiation of the MB solution containing the ZnO–ZnS composite rods synthesized with 1, 2, and 3 h sulfidation durations reached approximately 45.2%, 34.9%, and 21.6%, respectively. Notably, the decrease in the absorbance peak intensity in the UV region is associated with the mineralization of the MB dye during photodegradation.^15^ The substantial decrease in the absorbance peak intensity with the irradiation duration for the MB solution containing ZnO–ZnS composite rods synthesized with 3 h sulfidation in the UV region supported the observation of the superior discoloration ability of these ZnO–ZnS composite rods among the various composite rod photocatalysts tested. During photodegradation of a MB solution containing semiconductors, hydroxyl radicals produced in the solution are strong oxidizing agents and effectively decompose MB dyes.^33^ The photoexcited electrons or holes in the ZnO and ZnS semiconductors were transferred to the active surface where they participate in redox reactions with water molecules to produce a large number of hydroxyl radicals to decompose the MB dyes. The ZnS shell layer grown for the ZnO–ZnS composite rods obtained with 3 h sulfidation was well crystallized, and such a crystal structure achieved an efficient photoexcited charge separation through the heterointerface between the ZnO core and the ZnS shell of the composite rods, as shown in the PL results. Similarly, hydrothermally derived Bi_25_FeO_40_–Bi_2_WO_6_ heterostructures with close interfacial connections and good crystalline contacts at the heterojunction interface also exhibit an efficient photoexcited charge separation because of the matching band positions.^34^ The distinct granule-like surface features and the relatively high density of surface crystal defects obtained for the ZnS shell layer after 3 h sulfidation were beneficial to promote surface dye adsorption on the composite rods. Surface crystal defects in hydrothermally derived CdWO_4_–Bi_2_O_2_CO_3_ core–shell heterostructure photocatalysts help enhance solar light activity.^35^ Moreover, studies have shown that the photocatalytic efficiency of a CuBi_2_O_4_ photocatalyst is strongly affected by the surface morphology and a distinct extrusion of the surface structures shows satisfactory photodegradation activity for MB dyes.^36^ These factors accounted for the superior photoactivity observed for the ZnO–ZnS composite rods obtained after 3 h sulfidation. To further understand the role of active species in the photocatalytic processes, trapping experiments were carried out to detect the active components.^37–40^ In Fig. S2,† the photodegradation level of the MB solution decreases slightly after the addition of tertbutanol (TBA; a hydroxyl radical scavenger). Notably, the photodegradation of the MB solution was significantly inhibited after the addition of the benzoquinone (BQ; a superoxide anion radical scavenger) and ammonium oxalate (AO; a hole scavenger). This means that superoxide anion radicals and holes act as the main active species in the photocatalytic process herein. The stability of these ZnO–ZnS composite rods was further evaluated. Successive experimental cycles were conducted for the photocatalytic degradation of the MB solution, and the results are shown in Fig. 7(f). When the ZnS–ZnO-3 composite rods were used for the first time, approximately 78.4% of the MB solution was degraded after 75 min. The ZnO–ZnS rod catalysts were quite stable even after five cycles of photocatalytic degradation of the MB solution. After five cycles test, approximately 75.1% of the MB solution was degraded after 75 min, as shown in Fig. 7(f).

## Conclusions

Hydrothermally derived ZnO rods decorated with various crystalline features forming a ZnS shell layer were synthesized using a two-step hydrothermal methodology with 1, 2, and 3 h sulfidation durations. Increasing the sulfidation duration increased the crystallinity and granularity of the ZnS shell layer surface of the ZnO–ZnS core–shell composite rods. The longest sulfidation duration (3 h) resulted in a distinct granular ZnS shell layer. Moreover, our results demonstrate that the effective charge separation at the ZnO–ZnS heterointerface and an increased number of surface crystal defects in the ZnS shell layer were crucial for enhancing the photocatalytic performance of the ZnO–ZnS composite rods. A 3 h sulfidation process yielded a high degree of charge separation, which enabled these ZnO–ZnS composite rods to exhibit the highest photocatalytic activity for MB dye degradation.

## Conflicts of interest

There are no conflicts to declare.

## Supplementary Material

RA-008-C7RA13061A-s001
