# Supplementary material for: Surface crystal feature-dependent photoactivity of ZnO–ZnS composite rods via hydrothermal sulfidation
Source: RSC Adv. 2018 Jan 31;8(9):5063–70. doi: 10.1039/c7ra13061a (PMC9078038; doi:10.1039/c7ra13061a)
Supplement: RA-008-C7RA13061A-s001 [file RA-008-C7RA13061A-s001.pdf]

Electronic Supplementary Material (ESI) for RSC Advances.
This journal is © The Royal Society of Chemistry 2018

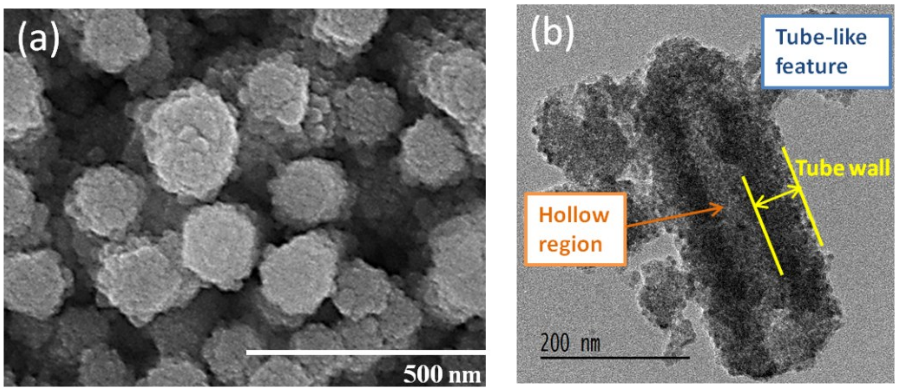

Fig. S1 (a) SEM image of the ZnO rods treated with 4-h sulfidation. (b) TEM image of the ZnO rod treated with 4-h sulfidation wherein the ZnO rod transformed into ZnS tube.

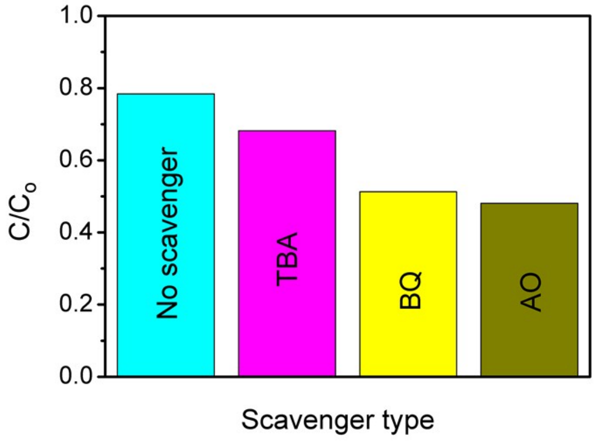

Fig. S2 The effects of various scavengers for the photocatalytic degradation of MB solution using ZnO-ZnS-3 for 75 min.
